# Regular Low-Intensity Exercise Prevents Cognitive Decline and a Depressive-Like State Induced by Physical Inactivity in Mice: A New Physical Inactivity Experiment Model

**DOI:** 10.3389/fnbeh.2022.866405

**Published:** 2022-05-06

**Authors:** Jimmy Kim, Jonghyuk Park, Toshio Mikami

**Affiliations:** ^1^Department of Anatomy and Neurobiology, Graduate School of Medicine, Nippon Medical School, Tokyo, Japan; ^2^Department of Health and Sports Science, Nippon Medical School, Tokyo, Japan

**Keywords:** physical inactivity, low-intensity exercise, cognitive function, depressive-like state, hippocampus

## Abstract

Regular exercise has already been established as a vital strategy for maintaining physical health *via* experimental results in humans and animals. In addition, numerous human studies have reported that physical inactivity is a primary factor that causes obesity, muscle atrophy, metabolic diseases, and deterioration in cognitive function and mental health. Regardless, an established animal experimental method to examine the effect of physical inactivity on physiological, biochemical, and neuroscientific parameters is yet to be reported. In this study, we made a new housing cage, named as the physical inactivity (PI) cage, to investigate the effect of physical inactivity on cognitive function and depressive-like states in mice and obtained the following experimental results by its use. We first compared the daily physical activity of mice housed in the PI and standard cages using the nano-tag method. The mice’s physical activity levels in the PI cage decreased to approximately half of that in the mice housed in the standard cage. Second, we examined whether housing in the PI cage affected plasma corticosterone concentration. The plasma corticosterone concentration did not alter before, 1 week, or 10 weeks after housing. Third, we investigated whether housing in the PI cage for 10 weeks affected cognitive function and depressive behavior. Housing in an inactive state caused a cognitive decline and depressive state in the mice without increasing body weight and plasma corticosterone. Finally, we examined the effect of regular low-intensity exercise on cognitive function and depressive state in the mice housed in the PI cage. Physical inactivity decreased neuronal cell proliferation, blood vessel density, and gene expressions of vascular endothelial growth factors and brain-derived neurotrophic factors in the hippocampus. In addition, regular low-intensity exercise, 30 min of treadmill running at a 5–15 m/min treadmill speed 3 days per week, prevented cognitive decline and the onset of a depressive-like state caused by physical inactivity. These results showed that our novel physical inactivity model, housing the mice in the PI cage, would be an adequate and valuable experimental method for examining the effect of physical inactivity on cognitive function and a depressive-like state.

## Introduction

Regular exercise has already been established as a vital strategy for maintaining physical health in humans and animals. Numerous human studies have reported that physical inactivity is a primary factor that causes obesity, muscle atrophy, and metabolic diseases (Booth et al., 2017; Bowden Davies et al., 2019). Moreover, physical inactivity (lack of exercise) is the fourth leading risk factor for death in the world after hypertension, diabetes, and smoking (Lee et al., 2012) and is linked to decreased cognitive function and a decline in mental health in older adults (Hamer and Stamatakis, 2014; Cunningham et al., 2020). In recent years, the increase in working from home and the suspension of sports facilities due to the infectious spread of the coronavirus disease (COVID-19) is increasing the number of patients with diseases related to physical inactivity worldwide. There have been only a few studies, however, investigating the mechanism underlying cognitive decline due to physical inactivity and the preventive effects of exercise against physical inactivity-induced cognitive decline.

Many previous studies investigating physical inactivity using animals targeted the muscle atrophy induced by the disuse of skeletal muscles rather than the lack of physical activity; in these studies, hindlimb suspension (Theilen et al., 2018), Gibbs fixation (Morimoto et al., 2013; Ye et al., 2013) and hindlimb nerve denervation (Lala-Tabbert et al., 2019) were used as the experimental methods for causing skeletal muscle atrophy. Such an experimental method can, indeed, cause skeletal muscle atrophy. It is important to note that these methods generally cause severe stress in animals and are not suitable for examining the effect of long-term physical inactivity on cognitive function and depressive states because stress usually leads to cognitive decline and depressive states.

Recently, two new experimental models have been reported to examine the physiological influence of physical inactivity. In one model, the investigators analyzed mice housed in a smaller cage than the standard cage that prevented climbing on the cage lid (Roemers et al., 2019). In another model, mice trained with a running wheel were subsequently denied exercise by removal of the running wheel (Nishijima et al., 2013). However, experimental conditions that induce physical inactivity in animals could simultaneously bring about isolation stress for animals. Furthermore, isolation stress causes cognitive decline (Rivera et al., 2020) and depressive disorder (Lee et al., 2020) in experimental animals. Therefore, isolation stress needs to be avoided to accurately examine the effect of physical inactivity on cognitive function and depressive disorder. With this in mind, the previous two studies did not take measures to avoid isolation stress.

Adult hippocampal neurogenesis is a phenomenon in which neurons are newly generated in the hippocampal dentate gyrus; the rate of hippocampal neurogenesis is decreased by aging (Yang et al., 2015; Horowitz et al., 2020) and chronic stress (Kiuchi et al., 2012; Bambico et al., 2015; Planchez et al., 2021) and is increased by regular exercise (Kiuchi et al., 2012; Choi et al., 2018) and antidepressant drugs (Jedynak et al., 2014). Furthermore, previous studies have shown that adult hippocampal neurogenesis occurs near the local microvasculature of the hippocampus (So et al., 2017) and that chronically stressed mice exhibit decreased capillary density in the hippocampal dentate gyrus (Kiuchi et al., 2012). Regarding the relationship between neurogenesis and blood vessels in the hippocampus, Fournier and Duman (2012) demonstrated that stress-induced decrease in hippocampal neurogenesis mainly occurred near capillaries and suggested that decreased blood flow due to chronic stress led to blood flow suppression and decreased capillary density in the hippocampus, followed by depression. These findings suggest that the development and improvement of a depressive state may be closely related to changes in neurogenesis and angiogenesis in the hippocampus. Moreover, vascular endothelial growth factor (VEGF) is critical in hippocampal neurogenesis and angiogenesis (Rich et al., 2017). A previous study showed that neurogenesis and angiogenesis were enhanced by antidepressant drugs and VEGF overexpression (Udo et al., 2008), whereas VEGF receptor inhibitors canceled the effect of antidepressant drugs (Warner-Schmidt and Duman, 2007; Greene et al., 2009), thus indicating that VEGF is necessary for neurogenesis, angiogenesis, cognitive maintenance, and antidepressant action. The effects of physical inactivity on adult hippocampal neurogenesis, angiogenesis, and VEGF have not yet been elucidated.

Here, we made a unique new cage, named as the physical inactivity (PI) cage using transparent acrylic plates to decrease the mice’s physical activity while reducing isolation stress as much as possible. Using the PI cages, we compared the amount of physical activity of the mice housed in the PI cages and the standard cages, examined the effect of physical inactivity on cognitive function and a depressive-like state, and analyzed the preventative effect of regular low-intensity exercise on behavioral alteration due to physical inactivity.

## Materials and Methods

### Animal and Ethical Approval

We conducted all experimental procedures and animal treatments according to the laboratory animal manual guidelines of Nippon Medical School. This study was approved by the Animal Care and Use Committee of Nippon Medical School (approval number was 28-023) and complies with animal research (ARRIVE) guidelines. Male C57BL/6J mice (10 weeks old, *n* = 73) were purchased from Sankyo Lab Service Co. (Tokyo, Japan) and used for all experiments. All mice were housed under 23 ± 2°C and with a 12-h light/dark cycle (light on 08:00–20:00). Standard chow (MF; Oriental Yeast Co., Ltd., Tokyo, Japan) and drinking water were supplied *ad libitum*.

### Inactivity Cage

**FIGURE 1:**
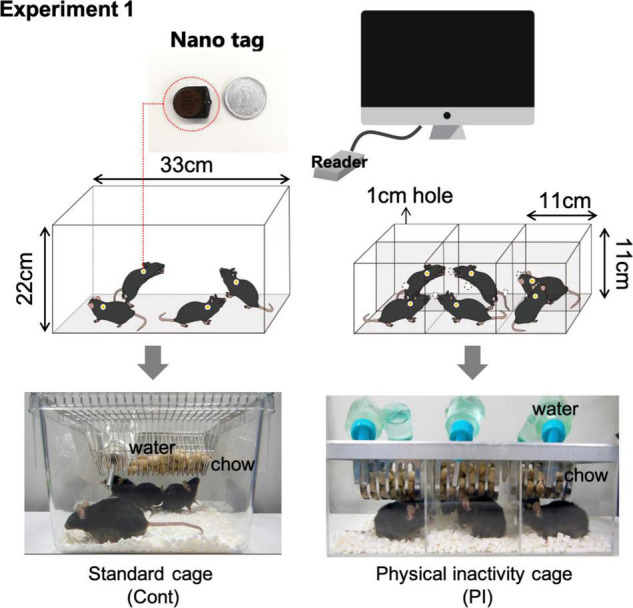
Measurement of the daily physical activity of mice using nano-tag apparatuses. Graphical explanation of measurement of the daily physical activity of mice using the nano-tag method.

Previous studies have shown that the mice’s regular cage (33 cm length, 22 cm height) was partitioned into six parts with aluminum boards to decrease the mice’s physical activity (Nakajima et al., 2009, 2010). For the present study, we made new cages to decrease the physical activity of the mice using acrylic plates and called the structure the physical inactivity (PI) cage (Figure 1A). In the PI cage, the residential space for a mouse was 11 cm in length and 11 cm in height, equal to that in the aluminum-divided cage; however, the PI cage differed from the aluminum-divided cage in the following two points. First, each mouse could visually see the mouse in the next compartment, as the cage was built using a transparent acrylic plate. Second, the mice could touch each other’s noses through a 12-mm hole drilled in the wall (Figure 1). When the mice are bred individually, they are usually exposed to isolation stress, which is caused by perturbing social interaction (Arzate-Mejia et al., 2020). Perturbation of social interaction would be caused by being visually isolated from other mice and losing physical contact with the other mice. Therefore, the PI cage was modified in two manners shown above. The housing contained food and water, so the mice could freely consume chow and tap water from the bait box and water bottle mounted on the cage ceiling, respectively.

### Experimental Design

**FIGURE 2:**
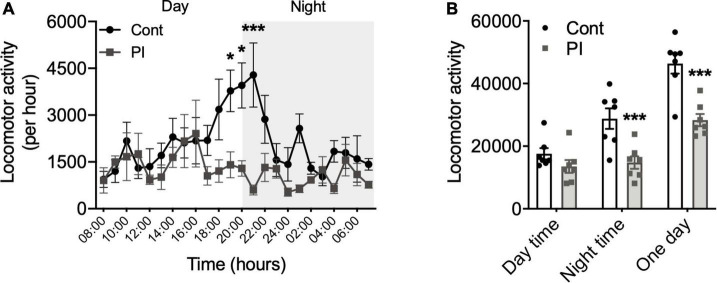
Comparison of the physical activity amount in the standard and PI cages. **(A)** Physical activity amount per hour during 1 day. **(B)** Physical activity amount during daytime (left), during nighttime (center), during 1 day (right). All data are presented as the mean ± SEM (Cont cage, *n* = 7; PI cage, *n* = 6). Data were analyzed using two-way ANOVA with Bonferroni’s *post-hoc* test **(A)**, and unpaired *t*-test analyzed data **(B)**. *p < 0.05, ***p < 0.001 in comparison with the Cont mice.

**FIGURE 3:**
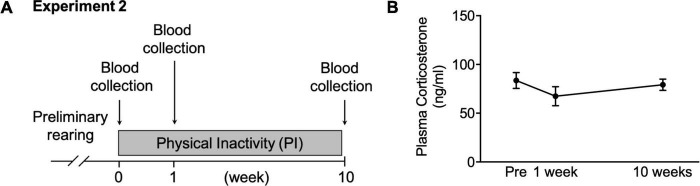
Changes in plasma corticosterone concentration during housing in the PI cage. **(A)** Schedule for collecting blood during housing in the PI cage. **(B)** Plasma corticosterone concentration before housing and at 1st and 10th weeks. All data are presented as the mean ± SEM (*n* = 10). Data were analyzed using one-way ANOVA with Dunnett’s *post-hoc* test.

**FIGURE 4:**
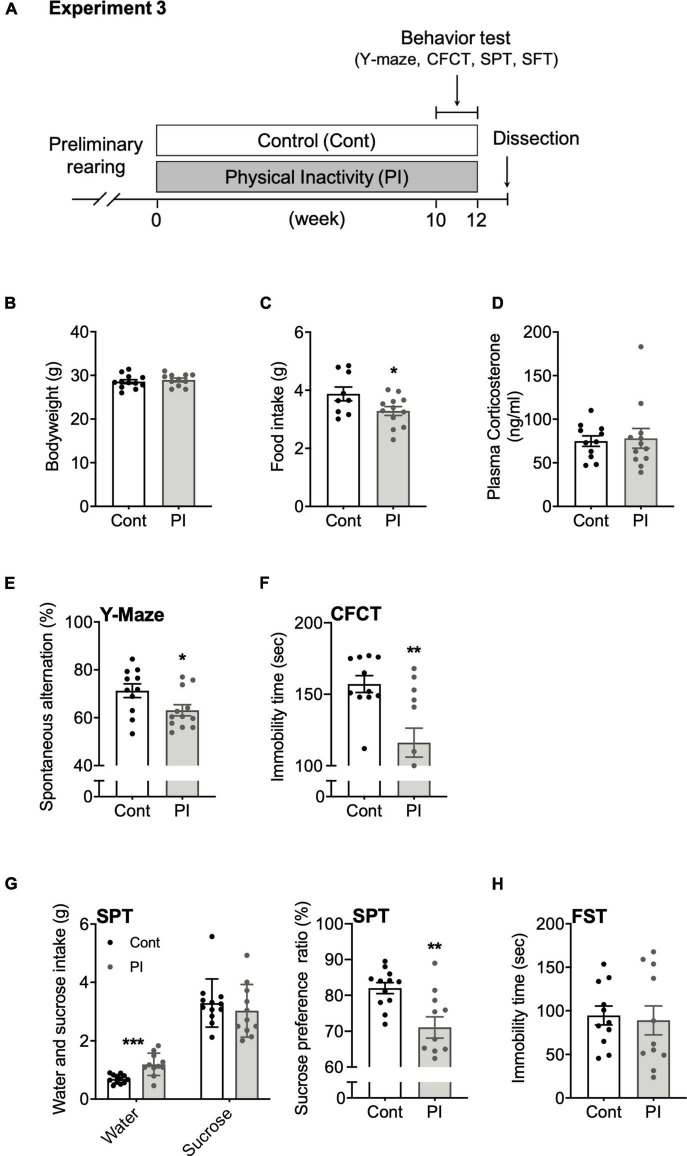
Housing in the PI cage induced cognitive decline and a depression-like state without increasing plasma corticosterone concentration. **(A)** Experimental protocol for physical inactivity housing for 10 weeks and behavioral tests. **(B)** Body weight. **(C)** Food intake on the 10th week. **(D)** Plasma corticosterone concentration. **(E)** Accuracy rate in the Y-maze test. **(F)** Immobility time in the contextual fear conditioning test (CFCT). **(G)** Sucrose preference ratio in the sucrose preference test (SPT). **(H)** Immobility time in the forced swim test (FST). All data are presented as the mean ± SEM (Control mice, *n* = 12; PI mice, *n* = 11). The Student’s unpaired *t*-test analyzed data. *p < 0.05, **p < 0.01 in comparison with the Cont mice.

**FIGURE 5:**
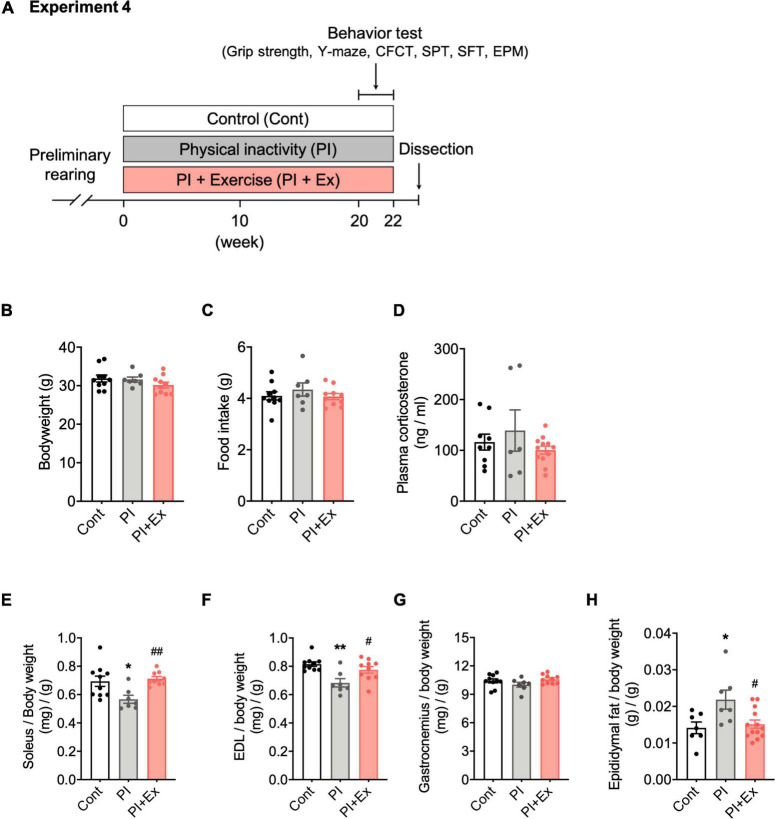
Regular low-intensity exercise prevented muscle weight loss and fat mass gain from housing in the PI cage. **(A)** Experimental design for physical inactivity housing for 20 weeks and cognitive testing. **(B)** Body weight. **(C)** Food intake on 20th week. **(D)** Plasma corticosterone concentration on 20th week. **(E–G)** Soleus, Extensor digitorum longus (EDL), and Gastrocnemius muscle weight normalized to body weight. **(H)** Epididymal fat weight normalized to body weight. All data are presented as the mean ± SEM (Cont mice, *n* = 10; PI mice, *n* = 7; PI + Ex mice, *n* = 10). Data were analyzed using one-way ANOVA with Tukey’s *post-hoc* test. *p < 0.05, **p < 0.01 compared with the Cont mice and *^#^p* < 0.05, *^##^p* < 0.01 compared with PI mice.

In the present study, we conducted the following four experiments. In experiment 1, we compared the physical activity of the mice housed in the PI cage and the standard mouse cage using a nano-tag (Figures 1, 2). Experiment 2 investigated whether housing mice in the PI cage affected their plasma corticosterone concentration (Figure 3). Experiment 3 investigated whether housing the mice in the PI cage for 10 weeks affected their cognitive function and depressive behaviors (Figure 4). Finally, experiment 4 investigated the effect of regular low-intensity exercise on cognitive function and depressive state in the mice housed in the PI cage for 20 weeks (Figure 5).

#### Experiment 1

##### Physical Activity Measurement

We compared the locomotor activity of the mice housed in the PI cage and standard mouse cage (Figure 1). For this purpose, we implanted nano-tags onto the back skin of the mice under anesthesia. Nano-tags (KISSEI COMTEC, Matsumoto, Japan) can be surgically implanted in a small laboratory animal body and quantify physical activity by measuring frequency and amount of vibration using a three-dimensional accelerometer inside them and recording the data in the device’s internal memory (Sakai et al., 2020). After a week’s recovery from surgery, the physical activity levels of the mice measured by the implanted nano-tag were recorded in the PI or the standard mouse cages for two consecutive days, respectively (Figure 1). Using nano-tags allowed locomotive levels in the standard cage to be measured when the subject mouse was residing with the other mice.

#### Experiment 2

##### Examination of Plasma Corticosterone During Long-Term Housing in the Physical Inactivity Cages

Mice were housed in the PI cage for 10 weeks, and blood was collected from a tail vein under gas anesthesia with isoflurane using a small animal anesthesia device (SN-487-0T; Sinano Mfg. Co., Ltd., Tokyo, Japan) (Figure 3A). Blood was collected before housing and at the first and tenth weeks of housing. Subsequently, blood was centrifuged to collect plasma which was stored at –80°C until analysis.

#### Experiment 3

##### Long-Term Housing in the Physical Inactivity Cages

Twenty-two mice were divided into control (Cont) mice and physical inactivity (PI) mice groups. In the former group, four mice were housed in each standard mouse cage, whereas in the latter, one mouse was housed in each compartment of the PI cage. Under each condition, the mice were housed for 10 weeks, then subjected to several behavioral tests to examine their cognitive function and depressive state. Two days after the last session of behavioral testing in Experiment 3, we dissected all the mice between 10 a.m. and 3 p.m. (Figure 4A). Plasma was isolated and stored at –80°C as described above.

#### Experiment 4

##### Long-Term Housing in the Physical Inactivity Cages With Regular Low-Intensity Exercise

Thirty-three mice were divided into three groups. (1) Control (Cont) mice: Four mice per cage were housed in a standard mouse cage. (2) Physical inactivity (PI) mice: housed in the PI cage. (3) Physical inactivity and exercised (PI + Ex) mice: housed in the PI cage and subjected to 30 min of treadmill running at a treadmill speed of 5–15 m/min, 3 days per week (Figure 5A). The treadmill speed was changed depending on the physical conditions of the mice on the exercise day. Twenty weeks later, all mice were examined for muscle strength, endurance exercise capacity, and behavioral tests to analyze the cognitive function and depressive state. On days two and three after the last session of behavioral tests in Experiment 4, we dissected all the mice between 10 a.m. and 3 p.m. as follows. First, the mice were anesthetized by intraperitoneal injection of three mixed anesthesia (containing Domitor, Midazolam, Bettlefar, and saline) at 0.75 mg/kg. Next, body weight was measured, blood was collected *via* heart puncture, and the heart was perfused with saline *via* the right ventral vein. We then collected the brain, epididymal fat, and skeletal muscles, including the soleus, extensor digitorum longus (EDL), and gastrocnemius samples. The brain was cut in half, and the hippocampus was separated from the right hippocampus. The brain’s left hemisphere was fixed for histochemical analysis as described below. The collected samples (plasma, hippocampus, soleus, gastrocnemius, EDL, and epididymal fat) were frozen in liquid nitrogen and stored at -80°C until analysis.

### Measurement of Muscle Strength and Endurance Exercise Capacity

We used a digital grip strength meter (GPM-100; Melquest, Japan) to measure forelimb or all-limb grip strength, based on the study of Takeshita et al. (2017); the apparatus measured the grip strength of the mice and showed the peak force strength (in grams). To measure the forelimb or all-limb grip strength, we had the mice grasp the device’s grip with their forelimb or all limbs and pulled the mouse tail from behind. The tension recorded by the gauge when the mice released their limbs from the bar was measured and expressed as grip strength (in grams). Results of three measurements per mouse were averaged and expressed as grip strength (gram per gram of body weight).

**FIGURE 6:**
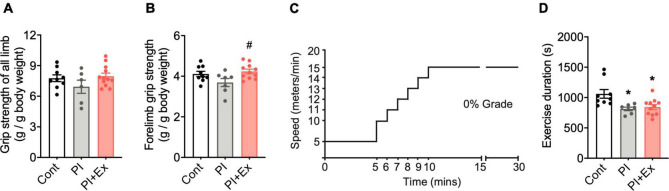
Regular low-intensity exercise prevents decreased muscle strength due to physical inactivity housing but shows no improvement in endurance exercise capacity. **(A,B)** Both all-limb and forelimb grip strengths normalized to body weight. **(C)** Protocol of endurance exercise test. **(D)** Time (seconds) that mice could run for until exhaustion in endurance exercise test on the treadmill. All data are presented as the mean ± SEM (Cont mice, *n* = 10; PI mice, *n* = 7; PI + Ex mice, *n* = 10). Data were analyzed using one-way ANOVA with Tukey’s *post-hoc* test. *p < 0.05 compared with the Control mice and *^#^p* < 0.05 compared with PI mice.

We also conducted a running test on a treadmill to examine the endurance exercise capacity of the mice according to the method of Rowe et al. (2013) with a slight modification. Before the endurance test, the control and PI mice, which did not undergo regular running on the treadmill, were acclimated to the exercise by running on the treadmill with no tilt angle for 10 min, at the speed of 12 m/min, three times a week for 1 week. In the endurance test, using the treadmill with no treadmill tilt angle, the treadmill speed was initially 5 m/min. After 5 min, the speed was subsequently increased by 1 m/min every 1 min to a maximum 15 m/min speed. After maximum speed was reached, the mice ran at the same speed until exhaustion (Figure 6C).

### Behavioral Tests

#### Y-Maze Test

We conducted a Y-maze test to examine working memory based on the study of Rubaj et al. (2003) with slight modifications. The Y-maze consisted of three equally spaced arms (height, 12 cm; width, 3 cm; length, 40 cm). Initially placed at the end of one arm, the mice freely traversed the apparatus while video-recorded for 8 min. Complete entry was determined when the mouse’s hind paws had entirely entered an arm of the maze. “Right” choice was defined as consecutive entries into the three different arms. Spontaneous alternation was calculated as the percentage of correct entries to the total number of entries using the following formula: percentage alternation (%) = (number of alternations/total number of arm entries) × 100 (%).

#### Contextual Fear Condition Test

We also performed a slightly modified contextual fear-conditioning test (CFCT), based on the method of Uwaya et al. (2016) to measure long-term memory using the foot shock system model (MK-450MSQ; Muromachi Kikai Co., Ltd., Japan). After being left in the test box for 2 min, the mice received three electric foot shocks (0.8 mA, 2-s duration) with a 2-min interval; they were kept in the test box for an additional minute and were returned to their home cages. On the following day, they were placed in the same test box for 5 min and video-recorded to measure immobility time. Immobility time was analyzed using the Smart 3.0 software (Panlab Inc., Spain).

#### Sucrose Preference Test

We performed the sucrose preference test (SPT) in the mice’s home cages to measure depressive-like state based on the method of Muto et al. (2014). First, all mice were acclimatized to two-bottle conditions for 2 days. Subsequently, the mice were provided with two bottles during nighttime (6 p.m. to 10 a.m.): One contained water, and another 1% sucrose, which allowed them to choose freely. Then, tests were conducted for 3 days, during which the bottle placements (i.e., left and right sides) were interchanged daily. Water and sucrose intake was measured by weighing the bottles before and after the test. Sucrose preference ratio was calculated as follows: Sucrose preference percentage (%) = (volume of sucrose intake/total volume of sucrose and water intake) × 100 (%).

#### Forced Swimming Test

We performed the forced swimming test (FST) according to Petit-Demouliere et al. (2005). The mice were placed in a water-filled cylinder (height, 20 cm; diameter, 15 cm; water temperature, 25°C) and video recorded for 6 min. In addition, immobility time, determined by the mice’s floating duration during the last 4 min of the test, was measured using analytical software (Smart 3.0, Panlab Inc., Spain).

#### Elevated Plus Maze Test

The elevated plus maze (EPM) was performed according to the method of Moon et al. (2014). The EPM apparatus comprised two open and two closed arms connected to a common central platform. A single pillar, 50 cm in height from the room floor, supported the arms and the central platform. Initially, the mice were placed at the center of the platform and allowed to explore both arms for 5 min. The number of times that the mouse entered each arm, determined by both forefeet entering an arm, and the amount of time they spent in each arm were measured using analytical software (Smart 3.0, Panlab Inc., Spain).

### Immunohistochemical Analysis

For the histochemical analysis, the right brain hemispheres were post-fixed in 4% paraformaldehyde at 4°C overnight. The brain was extracted and sectioned as previously described (Kiuchi et al., 2012). Ki-67 sections were incubated for 30 min with 3% hydrogen peroxide in methanol to block endogenous peroxidase activity. After washing with phosphate-buffered saline (PBS), the sections were exposed to heat (100°C) in 100 mM citric acid buffer (pH 6.0) for 30 min using a microwave for antigen retrieval, and the sections were incubated and then blocked with normal goat serum for 2 h. Next, the sections were incubated with primary rabbit polyclonal anti-Ki67 antibody (1:500; Abcam, Cambridge, United Kingdom) with gentle shaking at 4°C for two nights. After washing with PBS, the sections were incubated with goat anti-rabbit biotinylated IgG (1:100; Vector Laboratories, Burlingame, CA) for 2 h. Next, Ki67 sections were incubated with avidin-biotin-horseradish peroxidase complex (VECTASTAIN ABC Kit reagent; Vector Laboratories) for 2 h. Vascularization was examined using CD31 immunohistochemistry. After incubating the sections with 3% hydrogen peroxide for 30 min, and normal rabbit serum for 1 h, they were incubated with an anti-mouse CD31 monoclonal antibody (1:50; BD Pharmingen, CA) for 2 nights at 4°C. After washing with PBS, the sections were incubated with goat anti-rabbit biotinylated IgG (1:100; Vector Laboratories, United States) for 2 h at room temperature. Finally, Ki-67 and CD31 sections were washed with PBS and developed using 3,3′-diaminobenzidine for 2 min. The sections that reacted with antibodies were mounted, dehydrated, and coverslipped using Permount mounting medium. The number of Ki67-positive cells and surface capillary density of CD31 in the hippocampal dentate gyrus were counted using a Leica DM3000 microscope (Leica, Germany). The areas of the hippocampal dentate gyrus were also measured using NIH ImageJ software (NIH Image Engineering, Bethesda, MD, United States) and the cell density per mm^3^ calculated.

### Isolation of Total RNA and Real-Time Quantitative Reverse Transcriptase-Polymerase Chain Reaction

To measure the mRNA expression of hippocampal brain-derived neurotrophic factor (BDNF), VEGF, the frozen hippocampus and soleus muscle were homogenized on ice in TRIzol lysis reagent (Qiagen, Valencia, CA). Total RNA was extracted from the homogenate according to the manufacturer’s instructions. Total RNA was quantified by measuring the absorption at 260 and 260/280 nm ratio to assess concentration and purity. Complementary DNA was synthesized using 1 μg of total RNA in a 20-μl reaction with the ReverTra Ace™ qPCR RT Master Mix with gDNA Remover (FSQ-301; Toyobo, Osaka, Japan) according to the manufacturer’s instructions. Quantitative real-time PCR was performed with the SsoAdvanced Universal SYBR Green Supermix (Bio-Rad) and a CFX Connect Real-Time PCR System (Bio-Rad Laboratories, United States) to quantify the mRNA levels. Glyceraldehyde 3-phosphate dehydrogenase (GAPDH) primers amplified the endogenous control product. The mouse-specific primers used were as follows: VEGF: forward 5′-CGTTTAACTCAAGCTGCCTCGC-3′, reverse 5′-CTTCCAGGAGTACCCCGACGAGATA-3′; BDNF: forward 5′-TGCAGGGGCATAGACAAAAGG-3′, reverse 5′-CTTATGAATCGCCAGCCAATTCTC-3′; GAPDH: forward 5′-CATCACTGCCACCCAGAAGA-3′, reverse 5′-ATG TTCTGGGCAGCC-3′. The 2-ΔΔCT method was used to analyze relative mRNA expression values.

### Western Blot

To measure the VEGF content in the skeletal muscle, a portion of the gastrocnemius muscle was homogenized in RIPA lysis buffer [50 mM Tris-HCL buffer (pH 7.4); 150 mM NaCl; 1% Triton X-100; 0.5% Sodium Deoxy Cholate; 0.1% Sodium Dodecyl Sulfate] containing a proteinase inhibitor cocktail (Sigma-Aldrich, MI) and centrifuged at 14,000 × g for 10 min at 4°C. The supernatant’s protein concentration was determined using a BCA protein assay kit (Pierce). Aliquots (20 μg protein) were mixed with sodium dodecyl sulfate (SDS) sample buffer containing 1% mercaptoethanol, boiled for 5 min, and electrophoresed on 4–20% SDS polyacrylamide gradient gel. After electrophoresis, the proteins were blotted onto a polyvinylidene difluoride membrane at 20 mA for 60 min using the Bio-Rad Mini-PROTEAN gel system. After blotting, the membrane was washed with Tris-buffered saline (TBS) containing 0.1% Tween-20 (TBS-T) and blocked with TBS-T containing 5% skim milk for 1 h at room temperature. After washing with TBS-T, the membrane was probed with monoclonal anti-VEGFA20 antibody (1:200 diluted; Santa Cruz, United States) or monoclonal anti-GAPDH antibody (1:200 diluted; Santa Cruz) in TBS-T containing 3% skim milk at 4°C for 48 h with gentle shaking. Subsequently, after washing with TBS-T, the membrane was incubated with horseradish peroxidase-conjugated polyclonal rabbit anti-mouse IgG (1:3,000 diluted; Zymed, CA) in TBS-T containing 3% skim milk at room temperature for 2 h. Finally, the membrane was washed with TBS-T and developed using an ImmunoStar^®^ LD (FUJIFILM) Western blotting detection reagent (GE Healthcare, United Kingdom). The chemical luminescence of the membrane was detected using a C-DiGit™ system (Li-COR). Densitometric analysis was performed using Image Studio Digits ver. 4.0 (Li-COR). Relative protein expression was calculated by determining the ratio of each protein to GAPDH.

### Measurement of Plasma Corticosterone and Vascular Endothelial Growth Factor Concentration

Plasma corticosterone and VEGF concentrations were measured using the corticosterone enzyme-linked immunosorbent assay kit (Cayman Chem., MI, United States) or the mouse VEGF ELISA Kit (Proteintech, United States) according to the manufacturer’s instructions, respectively. In corticosterone ELISA, we measured each sample in duplicate using two plates, and the intra- and inter-assay coefficient of variations were 0.011 and 0.097, respectively. In VEGF ELISA, we measured each sample in duplicate using one plate, and the intra-coefficient of variations was 0.064.

### Statistical Analyses

Statistical analyses were performed using Prism version 8 (GraphPad Software Inc., San Diego, CA, United States). Values are expressed as mean ± standard error of the mean (SEM). Comparisons of the two groups were performed using an unpaired two-tailed Student’s *t*-test. Plasma corticosterone was analyzed using a one-way ANOVA followed by Dunnett’s multiple comparisons test for *post hoc* analysis. Locomotor activity was analyzed using repeated measures two-way ANOVA followed by Bonferroni’s multiple comparisons test for *post hoc* analysis. For experiment 4, a one-way ANOVA was used with Tukey’s test corrected for multiple comparisons test. Statistical significance was assumed at *p*-values of < 0.05.

## Results

### Comparison of the Physical Activity Amount in the Standard and Physical Inactivity Cages

We compared physical activity levels of mice that were housed in the standard and PI cages for 2 days *via* implanted nano-tag. Figure 2A shows the changes in physical activity levels per hour throughout 1 day. Physical activity per hour during the daytime was not significantly different in the two cages, whereas that of the nighttime was lower in the PI cages compared to the standard cage, and there were significant differences observed from 19 to 21 h (Figure 2A). Moreover, when the 1-day amount of physical activity was calculated separately into daytime and nighttime, that of the daytime was equal in two cages (Figure 2B), whereas that of the nighttime was significantly lower in the PI cage than in the standard cage (*p* < 0.001, Figure 2B).

### Plasma Corticosterone Concentration During Housing in the Physical Inactivity Cage

We measured plasma corticosterone concentration to examine stress state in the mice housed in the PI cage long-term. The plasma corticosterone concentration did not alter before, 1 week after, or ten weeks after housing (Figure 3B).

### Assessments of Cognitive Function and Depressive-Like State in Physical Inactivity Mice

For 10 weeks, we housed the mice in the PI cages to examine whether long-term housing in the PI cage affects cognitive function and depressive behaviors. Ten weeks of housing in the PI cage did not affect body weight (*p* = 0.55, Figure 4B) but significantly decreased food intake (*p* = 0.04, Figure 4C). Plasma corticosterone concentration measured at the housing end did not significantly differ between the two conditions (*p* = 0.81, Figure 4D). Interestingly, the PI mice showed a significant reduction in the spontaneous alternation ratio in the Y-maze test (*p* < 0.03, Figure 4E) and immobility time in the CFCT (*p* < 0.002, Figure 4F) than the Cont mice. Furthermore, the SPT result showed that the Cont and PI mice had no significant difference in sucrose intake (*p* = 0.46); however, the PI mice had a significantly higher intake of water (*p* < 0.001). Therefore, the PI mice’s preference ratio for sucrose was lower than that of the Cont mice (*p* < 0.003, Figure 4G). Incidentally, there was no difference in the immobility time in the FST between the two conditions (*p* = 0.78, Figure 4H). Details of the data discussed here are shown in Supplementary Table 1.

### Effect of Regular Low-Intensity Exercise on Muscle Weight, Fat Mass Gain, Muscle Strength, and Endurance Running Capacity in Physically Inactive Mice

We examined the effect of regular low-intensity exercise on the cognitive function and depressive-like state of the mice housed in the PI cage. No significant difference in body weight, [*F*_(2, 24)_ = 1.36, *p* = 0.28, Figure 5B] at the time of dissection, and average food intake [*F*_(2, 24)_ = 0.659, *p* = 0.53, Figure 5C], during the housing period, was observed among the Cont, PI and PI + Ex mice. Plasma corticosterone concentrations at the time of dissection were not significantly different among the three groups [*F*_(2, 24)_ = 0.962, *p* = 0.40, Figure 5D]. Soleus muscle weight significantly decreased in the PI mice than in the Cont and PI + Ex mice [*F*_(2, 23)_ = 6.411, *p* = 0.01, Figure 5E], whereas no significant difference was noted between the Cont and PI + Ex mice. The extensor digitorum longus (EDL) muscle weight significantly decreased in the PI mice than in the Cont and PI + Ex mice [*F*_(2, 24)_ = 8.142, *p* = 0.002, Figure 5F], whereas no significant difference in gastrocnemius muscle weight was observed among the three groups [*F*_(2, 24)_ = 1.880, *p* = 0.17, Figure 5G]. In contrast, epididymal fat weight significantly increased in the PI mice than in the other two groups of mice [*F*_(2, 24)_ = 5.334, *p* = 0.01, Figure 5H]. Moreover, no significant differences in grip strength using all limbs were found among the three groups [*F*_(2, 24)_ = 1.623, *p* = 0.22, Figure 6A]. For grip strength using the forelimbs, the PI mice showed a significantly lower level than the PI + Ex mice [*F*_(2, 24)_ = 3.789, *p* = 0.04, Figure 6B]. The PI and PI + Ex mice showed a significantly shorter running time than the Cont group [*F*_(2, 24)_ = 6.493, *p* = 0.006, Figure 6D], whereas no significant difference was observed between the PI and PI + Ex mice. Details of the data mentioned here are shown in Supplementary Table 2.

### Regular Low-Intensity Exercise Prevented Cognitive Decline and Depression-Like State *via* Physical Inactivity

**FIGURE 7:**
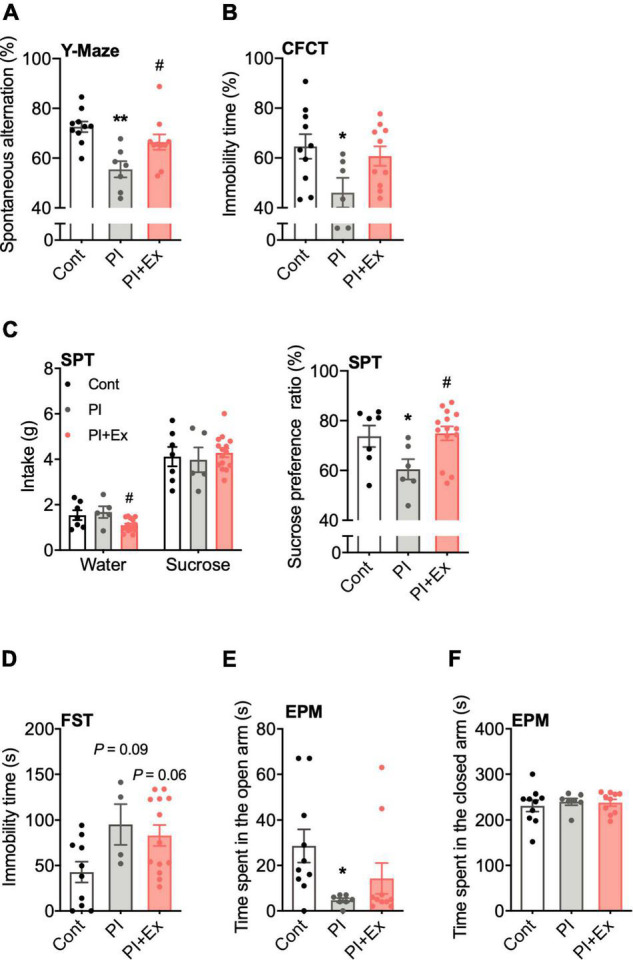
Regular low-intensity exercise prevents cognitive decline and depressive state due to physical inactivity. **(A)** Accuracy rate in the Y-maze test. **(B)** Immobility time in the contextual fear conditioning test (CFCT). **(C)** Sucrose preference ratio in the sucrose preference test (SPT). **(D)** Immobility time in the forced swim test (FST). **(E,F)** Time spent in the open and closed arm in the elevated plus-maze test (EPM). All data are presented as the mean ± SEM (Cont mice, *n* = 10; PI mice, *n* = 7; PI + Ex mice, *n* = 10). Data were analyzed using one-way ANOVA with Tukey’s *post-hoc* test. *p < 0.05, **p < 0.01 in compared with the Cont mice and *^#^p* < 0.05 in comparison with the PI mice.

We conducted four behavioral tests to examine the cognitive function and depressive-like state in mice. First, in the Y-maze test for examining working memory, the spontaneous alternation ratio was significantly lower in the PI mice than in the Cont and PI + Ex mice, and there was no difference between the Cont and PI + Ex mice [*F*_(2, 24)_ = 8.301, *p* = 0.002, Figure 7A]. Second, immobility time (%) in the CFCT for examination of long-term memory was significantly decreased in the PI mice than in the Cont mice [*F*_(2, 23)_ = 3.351, *p* = 0.05, Figure 7B]. Third, in the SPT to examine the depressive-like state in the mice, the SPT result showed that the Cont and PI mice had no significant difference in sucrose intake [*F*_(2, 24)_ = 0.263, *p* = 0.77]; however, the PI mice had a significantly higher intake of water [*F*_(2, 24)_ = 4.660, *p* < 0.02]. Therefore, the preference ratio for sucrose of the PI mice was lower than that of the Cont mice [*F*_(2, 24)_ = 4.091, *p* < 0.03, Figure 7C]. In the FST to examine depressive state, the Cont mice showed lower immobility time than the PI and PI + Ex mice, but the difference was not significant [*F*_(2, 24)_ = 3.802, *p* = 0.04, Figure 7D]. Lastly, in the EPM for examining anxiety, the time spent in the open arm significantly decreased in the PI mice than in the Cont mice [*F*_(2, 24)_ = 3.320, *p* = 0.05, Figure 7E]. However, the closed arm yielded no differences among the three groups [*F*_(2, 24)_ = 0.211, *p* = 0.81, Figure 7F]. Details of the data shown here are shown in Supplementary Table 3.

### Regular Low-Intensity Exercise Prevented the Deterioration of Ki-67 Positive Cells and Surface Capillary Density by Physical Inactivity Housing

**FIGURE 8:**
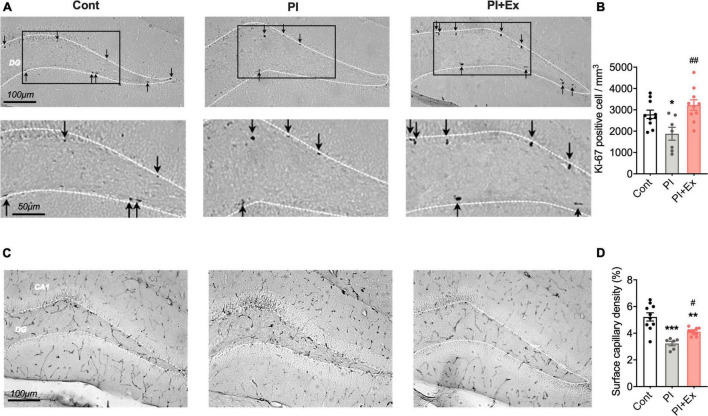
Regular low-intensity exercise prevents the decrease in Ki-67 positive cells and surface capillary density in the hippocampus due to physical inactivity housing. **(A,B)** Ki-67 positive cells in the hippocampus dentate gyrus. **(C,D)** Surface capillary density measured by CD31-positive cells in the hippocampus. All data are presented as the mean ± SEM (Cont mice, *n* = 10; PI mice, *n* = 7; PI + Ex mice, *n* = 10). Data were analyzed using one-way ANOVA with Tukey’s *post-hoc* test. *p < 0.05, **p < 0.01, ***p < 0.001 in comparison with the Cont mice and ^#^*p* < 0.05, ^##^*p* < 0.01 in comparison with the PI mice.

We measured the number of Ki-67 positive cells in the hippocampus to examine hippocampal neuronal cell proliferation. The number of Ki-67 positive cells in the PI + Ex mice was significantly higher than that in the PI mice, with no significant difference compared with the Cont mice [*F*_(2, 24)_ = 6.965, *p* < 0.004, Figures 8A,B]. Similarly, the number of Ki-67 positive cells in the PI mice was significantly lower than that in the Cont mice. Hippocampal surface capillary density, measured based on CD31-positive cells, also significantly decreased in the PI mice than in the Cont mice; the PI + Ex mice showed significantly higher levels of the PI mice [F(_2, 23)_ = 17.68, *p* < 0.001, Figures 8C,D].

### Regular Low-Intensity Exercise Increased Plasma Vascular Endothelial Growth Factor Concentration and Hippocampal Brain-Derived Neurotrophic Factor and Vascular Endothelial Growth Factor mRNA Expression

**FIGURE 9:**
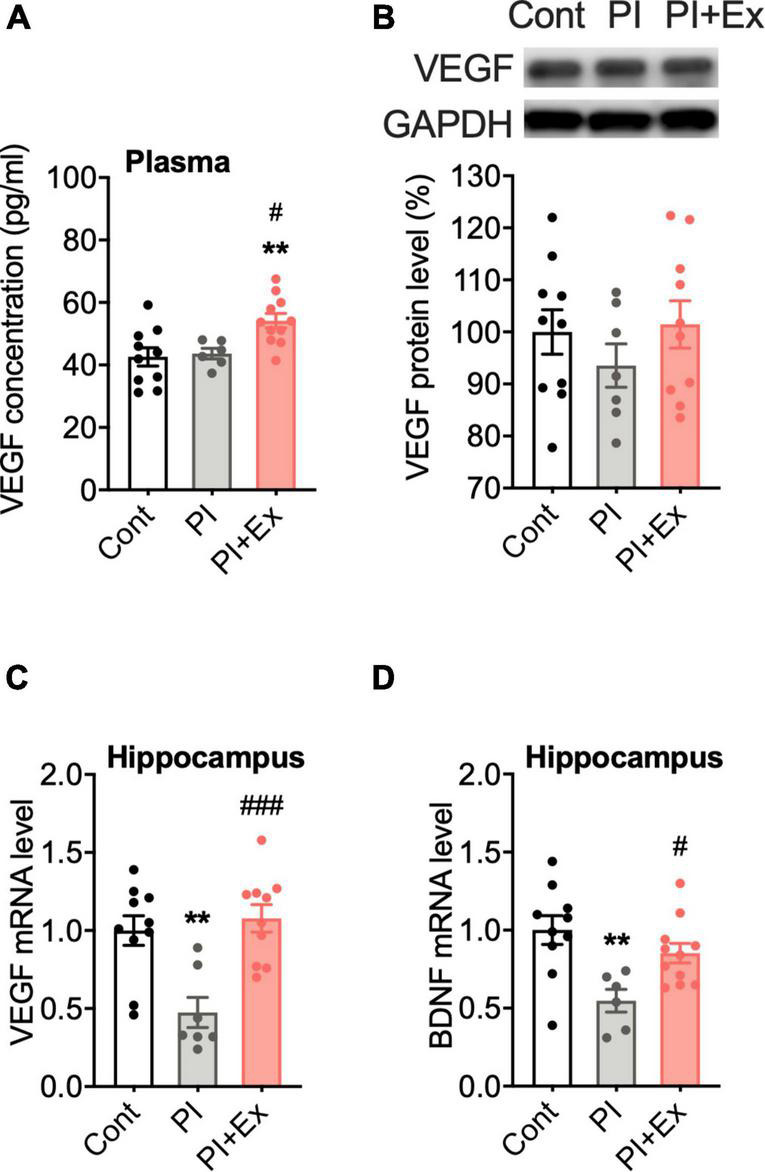
Regular low-intensity exercise increased plasma VEGF concentration and hippocampal BDNF and VEGF mRNA expression. **(A)** Plasma VEGF concentration. **(B)** Representative gel image of western blot analysis for VEGF protein expression in the gastrocnemius muscle. **(C)** Relative mRNA level for VEGF in the hippocampus. **(D)** Relative mRNA level for BDNF in the hippocampus. All data are presented as the mean ± SEM (Cont mice, *n* = 10; PI mice, *n* = 7; PI + Ex mice, *n* = 10). Data were analyzed using one-way ANOVA with Tukey’s *post-hoc* test. **p < 0.01, in comparison with the Control mice and ^#^*p* < 0.05, ^###^*p* < 0.001 in comparison with the PI mice.

Our immunohistochemical results showed that physical inactivity resulted in cognitive decline and a depressive-like state with decreased neuronal cell proliferation and angiogenesis in the hippocampus, which is consistent with a previous study result showing that hippocampal neurogenesis and angiogenesis are closely related (Heine et al., 2005). One of the contributing factors to hippocampal angiogenesis is VEGF; therefore, we examined changes in VEGF expression in the mice groups. Plasma VEGF concentration was significantly higher in the PI + Ex mice than in the other two groups of mice [*F*_(2, 24)_ = 6.897, *p* < 0.004, Figure 9A]. VEGF protein levels in the gastrocnemius muscle did not differ among the three groups [*F*_(2, 24_ = 0.7854, *p* = 0.47, Figure 9B]. Hippocampal VEGF mRNA expression significantly decreased in the PI mice than in the Cont and PI + Ex mice [*F*_(2, 24)_ = 10.54, *p* = 0.001, Figure 9C]; no significant difference was evident between the Cont and PI + Ex mice. The same pattern was observed for hippocampal BDNF mRNA expression [*F*_(2, 24_ = 6.745, *p* = 0.005, Figure 9D].

## Discussion

One of the purposes of this study was to establish a valid experimental model to examine the effect of long-term physical inactivity on cognitive function and a depressive-like state. For this purpose, we made a special cage using acrylic plates, which we named as the physical inactivity (PI) cage. Housing the mice in the PI cage decreased the space in which the mice could freely move, and measurements of the mice’s locomotor activity using a nano-tag showed that mice that were housed in the PI cage decreased nighttime physical activity to about 50% of those housed in a standard cage (Figure 2B). In addition, to measure physical activity, the mice were abdominally implanted with a nano-tag. Although a nano-tag is only about 3 g, it is about 1/10 of the mouse’s body weight. Therefore, the implantation of the nano-tag weighing 3 g could have restricted physical movement and lowered the mice’s physical activity; consequently, the mice’s amount of physical activity while housed in the standard cage could have been somewhat lower than that of the mice not implanted with a nano-tag. Thus, the decrease in physical activity by housing in PI cage could have been more than 50%. Therefore, these results showed that the size of the space in which the mice are daily residents is a limiting factor for the physical activity of the mice, and we think that housing the mice in the PI cage is a valuable way to cause physical inactivity in mice.

In the present study, the plasma corticosterone concentration of the mice housed in the PI cage did not significantly change during 10 weeks, from the start of housing to its end (Figure 3B). Moreover, no increase in plasma corticosterone concentration was observed even in the other two experiments (Figures 4D, 5D). Plasma corticosterone concentration is a primary indicator of stress state; therefore, this result suggests that housing in the PI cage would not cause severe stress to the mice. The reason that isolation stress during housing in the PI cage was alleviated is speculated as follows. First, the mice could visually confirm other mice next to them through a transparent acrylic wall (Figure 1). Second, they could touch their noses through holes drilled in the wall (Figure 1). It is important to note that this study did not measure stress indications other than plasma corticosterone concentration, such as plasma ACTH concentration (Lightman et al., 2020) and immunohistochemical analysis of Iba-1-positive cells in the hippocampal dentate gyrus (Rivera et al., 2020). Therefore, we need to clarify the existence and absence of the stress of the mice housed in the PI cage by measuring other stress indicators in future studies.

Long-term housing in the PI cage caused loss of muscle weight (soleus and EDL muscles) and epididymal fat mass gain (Figures 5E,F,H), whereas body weight and gastrocnemius muscle weight were not altered (Figures 5B,G). Moreover, it resulted in a reduction in muscle strength (not significant) (Figures 6A,B) and endurance exercise capacity (Figure 6D). In contrast previous studies reported that hindlimb suspension (Gaignier et al., 2014; Marzuca-Nassr et al., 2019) or cast fixation (Lang et al., 2012; Morimoto et al., 2013; Ye et al., 2013), even within 1–2 weeks, resulted in increased plasma corticosterone levels (Steffen and Musacchia, 1987) and atrophy of all muscles. Judging from these results, hindlimb or cast fixation results in quick disuse muscle atrophy with severe stress in a short-term period, whereas physical inactivity due to housing in the PI cage long-term would result in mild disuse muscle atrophy, energy consumption reduction, and functional deterioration of muscles. Furthermore, the former models provide severe stress to animals; therefore, these are considered inappropriate for examining the independent influence of physical inactivity on cognition decline and depression. Additionally, our experimental model would be a valuable strategy for investigating physical inactivity’s physiological and neurological influence on biogenic function, but we examined only muscle weight in the present study and did not study other indicators such as cross-sectional muscle area or muscle fiber composition. Therefore, we need to elucidate whether housing in the PI cage would result in muscle atrophy by measuring indicators other than muscle weight.

Previous studies have suggested that hippocampal neuronal proliferation decreases are closely related to cognitive decline *via* aging (van Praag et al., 2005; Horowitz et al., 2020) or chronic stress (Nakajima et al., 2009; Muto et al., 2014; Lee et al., 2018). Furthermore, chronic stress causes a decrease in hippocampal blood vessel density, which leads to a depressive-like state (Kiuchi et al., 2012). In the present study, long-term physical inactivity also caused cognitive decline and a depressive-like state, and the hippocampus’s decreased neurogenesis and vascular density were simultaneously observed. Therefore, we expected that the cause of this behavioral deterioration would be due to decreased neurogenesis and decreased vascular density in the hippocampus. The following two points were expected as the factors that triggered these hippocampal changes. The first is psychological stress induced by limiting the amount of physical activity. There is a possibility that psychological stress could result from the mice being unable to move as desired. The second was a reduction in the amount of myokines released from skeletal muscles due to the decreased use of these muscles from physical inactivity. Myokines, especially irisin, are re-released from skeletal muscles, and their release is increased by exercise (Jedrychowski et al., 2015) and contributes to improving cognitive function (Lourenco et al., 2019). The decreased use of skeletal muscle due to housing in the PI cages might have suppressed myokines released from skeletal muscles, but this study has not verified this point. The purpose of this study was to uncover the direct influences of physical inactivity on cognitive function and depressive-like state; however, at this moment, we can only show results including the influence of some additional stress.

Regular low-intensity exercise prevents the suppression of neuronal cell proliferation and angiogenesis in the hippocampus due to long-term physical inactivity, preventing cognitive decline and depressive behaviors. Newly generated neuronal cells are observed mainly near the capillaries in the hippocampus (Heine et al., 2005), and hippocampal neurogenesis and angiogenesis are closely related to each other (Heine et al., 2005). Furthermore, a previous study showed that regular, moderate exercise prevented neuronal cell proliferation and angiogenesis in the hippocampus (Kiuchi et al., 2012). Based on these results, we hypothesized that regular low-intensity exercise could prevent cognitive decline and the onset of a depressive-like state by inhibiting the decrease in neuronal cell proliferation and angiogenesis due to long-term physical inactivity.

In this study, plasma VEGF concentration and hippocampal VEGF mRNA expression were increased in the PI + Ex mice (Figures 7A,C). In a previous study, VEGF-overexpressing transgenic mice showed enhanced hippocampal neurogenesis (Udo et al., 2008), and intraventricular VEGF administration could also enhance hippocampal neurogenesis levels (Sun et al., 2003, 2006; Licht et al., 2011). Conversely, both hippocampus-specific VEGF knockdown (Sun et al., 2006) and VEGF receptor antagonist-administered mice (Jin et al., 2002) showed decreased hippocampal neurogenesis. Our previous study also showed that administration of the VEGF receptor antagonist SU1498 inhibits the increase in hippocampal cell proliferation resulting from regular exercise training (Kiuchi et al., 2012). The origin of plasma VEGF is likely the skeletal muscle and liver. The VEGF in skeletal muscle did not differ among the three groups in our study (Figure 7B). Moreover, regardless of the intensity, hepatic blood flow would decrease (Dyke et al., 1998). We expected that the decreased hepatic blood flow would decrease liver oxygen levels, followed by hypoxia-induced factor-1 and VEGF expression. Nevertheless, we could not examine the hepatic VEGF content as we did not collect liver samples.

A decrease in hippocampal BDNF results in cognitive decline and depressive-like state, whereas hippocampal BDNF recovery through several methods, including regular exercise, drug administration, and transgenic modification, restores cognitive function. In this study, the decreased hippocampal BDNF mRNA expression due to physical inactivity was restored by regular exercise (Figure 7D). This result indicates that physical inactivity causes cognitive decline and a depressive-like state by decreasing neuronal cell proliferation and BDNF expression in the hippocampus. In contrast, regular exercise prevents decreased hippocampal BDNF expression and thus maintains hippocampal cell proliferation and cognitive function.

### Limitations

The purpose of this study was to explore the direct influences of physical inactivity on cognitive function and a depressive-like state; however, at this moment, we can only show results including the influence of some external stress. In animal experiments, it is difficult to investigate the effect of physical inactivity on cognitive function and a depressive-like state without causing the experimental animals stress, especially isolation stress. To achieve the purpose, we will need further to alleviate the influence of isolation stress in the future. For this purpose, based on the result of the diurnal rhythm of the physical activity obtained, the cage size did not affect the daytime physical activity; therefore, to exclude isolation stress, it may be valid to rear mice as a group in a standard cage during the day and separately in the PI cages during nighttime. Furthermore, to distinguish the effect of the isolation stress and physical inactivity, it will need to compare the stress markers and behavioral changes between the mice housed in the PI cage and the mice housed separately. It also will be necessary to compare the effect of cage size on the changes in stress markers and behaviors. In addition, we also need to examine stress markers other than plasma corticosterone. Moreover, we suppose that the primary factor in inducing cognitive decline and onset of a depressive-like state due to physical inactivity is likely the reduction of myokines released from skeletal muscles resulting from skeletal muscle disuse. An experiment using neutralizing antibodies for irisin will be necessary to test this hypothesis. The present study is the first step in investigating the direct influences of physical inactivity on cognitive function and depressive-like state.

## Conclusion

To investigate the effect of physical inactivity on cognitive function and depressive-like state, we housed mice in the PI cage. Daily physical activity was decreased in the PI mice to about 50% of the control mice. Housing in the PI cage long-term resulted in cognitive decline and a depressive-like state with reduced hippocampal neuronal cell proliferation, hippocampal blood density, plasma VEGF level, hippocampal VEGF, and BDNF mRNA expression. Regularly low-intensity exercise restored the decreased hippocampal factors, preventing cognitive decline and a depressive-like state. The physical inactivity model *via* housing in the PI cage showed in the present study may become an adequate and valuable experimental model for investigating the effect of physical inactivity on brain function, particularly cognitive function.

## Data Availability Statement

The raw data supporting the conclusions of this article will be made available by the authors, without undue reservation.

## Ethics Statement

The animal study was reviewed and approved by the Animal Care and Use Committee of Nippon Medical School (approval no. 28-023).

## Author Contributions

TM and JK designed the study and wrote the manuscript. TM, JK, and JP performed the research and analyzed the data. All authors contributed to the article and approved the submitted version.

## Conflict of Interest

The authors declare that the research was conducted in the absence of any commercial or financial relationships that could be construed as a potential conflict of interest.

## Publisher’s Note

All claims expressed in this article are solely those of the authors and do not necessarily represent those of their affiliated organizations, or those of the publisher, the editors and the reviewers. Any product that may be evaluated in this article, or claim that may be made by its manufacturer, is not guaranteed or endorsed by the publisher.
