# Supplementary material for: Regular Low-Intensity Exercise Prevents Cognitive Decline and a Depressive-Like State Induced by Physical Inactivity in Mice: A New Physical Inactivity Experiment Model
Source: Front Behav Neurosci. 2022 May 6;16:866405. doi: 10.3389/fnbeh.2022.866405 (PMC9121131; doi:10.3389/fnbeh.2022.866405)

Supplementary Material

| **Group** | **Control (Cont)** | | | |  | **Physical inactivity (PI)** | | | | **t** | **df** | ***p*** |
| --- | --- | --- | --- | --- | --- | --- | --- | --- | --- | --- | --- | --- |
|  | **Mean** | **SEM** | **95% CI** | |  | **Mean** | **SEM** | **95% CI** | |  |  |  |
|  |  |  | **Lower** | **Upper** |  |  |  | **Lower** | **Upper** |  |  |  |
| **Bodyweight (g)** | 28.57 | 0.45 | 27.57 | 29.57 |  | 28.95 | 0.42 | 28.00 | 29.89 | 0.606 | 21 | .5513 |
| **Food intake (g)** | 3.88 | 0.24 | 3.33 | 4.43 |  | 3.29^*^ | 0.15 | 2.95 | 3.62 | 2.173 | 19 | .0426 |
| **Plasma corticosterone (ng/mL)** | 74.91 | 5.98 | 61.58 | 88.24 |  | 78.08 | 11.25 | 53.31 | 102.90 | 0.243 | 21 | .8108 |
| **Y-maze (%)** | 71.28 | 2.88 | 64.86 | 77.70 |  | 63.11^*^ | 2.32 | 58.00 | 68.22 | 2.277 | 21 | .0370 |
| **Contextual Fear Condition Test (s)** | 157.20 | 5.89 | 144.00 | 170.30 |  | 116.20^**^ | 10.18 | 93.77 | 138.60 | 3.404 | 21 | .0027 |
| **Sucrose Preference Test** | |  |  |  |  |  |  |  |  |  |  |  |
| **Water intake (g)** | 0.69 | 0.04 | 0.60 | 0.79 |  | 1.19^***^ | 0.16 | 0.93 | 1.44 | 4.168 | 21 | .0004 |
| **Sucrose intake (g)** | 3.29 | 0.24 | 2.76 | 3.82 |  | 3.02 | 0.27 | 2.42 | 3.63 | 0.744 | 21 | .4649 |
| **Sucrose Preference ratio (%)** | 82.01 | 1.54 | 78.62 | 85.40 |  | 71.05^**^ | 2.93 | 64.51 | 77.58 | 3.390 | 21 | .0028 |
| **Forced Swimming Test (sec)** | 94.56 | 10.84 | 70.40 | 118.70 |  | 89.05 | 16.53 | 52.22 | 125.90 | 0.279 | 20 | .7830 |

**Table S1.** Data on bodyweight, plasma corticosterone and behavior test in the Cont and PI groups

All data are presented as the mean ± SEM (Control mice, *n*=12; PI mice, *n*=11). The Student’s unpaired t-test analyzed data. *^*^p* < 0.05, *^**^p* < 0.01, *^***^p* < 0.001 in comparison with the Cont mice.

**Table S2.** One-way ANOVA statistical results for bodyweight, tissue mass, muscle strength, and endurance exercise capacity

| **Group** | **Control (Cont)** | |  | **Physical inactivity (PI)** | |  | **Physical inactivity + Exercise (PI+Ex)** | | **F** | **df** | ***p*** |
| --- | --- | --- | --- | --- | --- | --- | --- | --- | --- | --- | --- |
|  | **Mean** | **SEM** |  | **Mean** | **SEM** |  | **Mean** | **SEM** |  |  |  |
| **Bodyweight (g)** | 31.87 | 0.90 |  | 31.60 | 0.67 |  | 30.17 | 0.74 | 1.363 | 2,24 | .2751 |
| **Food intake (g)** | 4.10 | 0.16 |  | 4.34 | 0.26 |  | 4.07 | 0.12 | 0.659 | 2,24 | .5263 |
| **Plasma corticosterone (ng/mL)** | 116.30 | 15.54 |  | 139.1 | 40.61 |  | 100.50 | 7.80 | 0.962 | 2,24 | .3966 |
| **Soleus (mg) / bodyweight (g)** | 0.694 | 0.036 |  | 0.567^*^ | 0.029 |  | 0.712^##^ | 0.016 | 6.411 | 2,23 | .0061 |
| **EDL (mg) / bodyweight (g)** | 0.816 | 0.016 |  | 0.683^**^ | 0.030 |  | 0.775^#^ | 0.023 | 8.142 | 2,24 | .0020 |
| **Gastrocnemius (mg) / bodyweight (g)** | 10.42 | 0.218 |  | 10.01 | 0.255 |  | 10.59 | 0.148 | 1.880 | 2,24 | .1744 |
| **Epididymal (g) /bodyweight (g)** | 0.014 | 0.002 |  | 0.022 | 0.003 |  | 0.015 | 0.001 | 5.334 | 2,24 | .0121 |
| **All-limb grip strength (g) / bodyweight (g)** | 7.76 | 0.33 |  | 6.93 | 0.65 |  | 7.96 | 0.30 | 1.623 | 2,24 | .2183 |
| **Forelimb grip strength (g) / bodyweight (g)** | 4.11 | 0.13 |  | 3.70 | 0.20 |  | 4.24 | 0.11 | 3.789 | 2,24 | .0372 |
| **Endurance exercise capacity (sec)** | 1061.00 | 71.90 |  | 813.00 | 28.78 |  | 843.80 | 40.66 | 6.493 | 2,24 | .0056 |

All data are presented as the mean ± SEM (Cont mice, *n*=10; PI mice, *n*=7; PI+Ex mice, *n*=10). Data were analyzed using one-way ANOVA with Tukey’s *post hoc* test. **p* < 0.05, ***p* < 0.01 compared with the Cont mice and *^#^p* < 0.05, *^##^p* < 0.01 compared with PI mice.

| **Group** | **Control (Cont)** | |  | **Physical inactivity (PI)** | |  | **Physical inactivity + Exercise (PI+Ex)** | | **F** | **df** | ***p*** |
| --- | --- | --- | --- | --- | --- | --- | --- | --- | --- | --- | --- |
|  | **Mean** | **SEM** |  | **Mean** | **SEM** |  | **Mean** | **SEM** |  |  |  |
| **Y-Maze (%)** | 72.59 | 2.15 |  | 55.50^**^ | 3.26 |  | 66.44^#^ | 3.13 | 8.301 | 2,24 | .0018 |
| **Contextual Fear Condition Test (%)** | 64.61 | 4.91 |  | 46.07^*^ | 5.96 |  | 60.74 | 3.93 | 3.351 | 2,23 | .0528 |
| **Sucrose Preference Test** |  |  |  |  |  |  |  |  |  |  |  |
| **Water intake (g)** | 1.54 | 0.22 |  | 1.67 | 0.25 |  | 1.10 | 0.07 | 4.660 | 2,24 | .0195 |
| **Sucrose intake(g)** | 4.12 | 0.43 |  | 4.00 | 0.55 |  | 4.30 | 0.18 | 0.263 | 2,24 | .7709 |
| **Sucrose Preference Test (%)** | 73.75 | 4.30 |  | 60.48^*^ | 4.08 |  | 74.91^#^ | 2.81 | 4.091 | 2,24 | .0296 |
| **Forced Swimming Test (sec)** | 42.83 | 11.49 |  | 95.17 | 22.35 |  | 83.11 | 11.46 | 3.802 | 2,24 | .0368 |
| **Elevated Plus Maze Test**  **Time spent in the open arm (s)** | 28.60 | 7.29 |  | 4.71* | 0.92 |  | 14.30 | 6.77 | 3.320 | 2,24 | .0533 |
| **Elevated Plus Maze Test**  **Time spent in the closed arm (s)** | 231.20 | 12.66 |  | 240.00 | 7.36 |  | 238.00 | 7.47 | 0.211 | 2,24 | .8110 |

**Table S3.** One-way ANOVA statistical results for the behavior test in Cont, PI and PI+Ex groups

All data are presented as the mean ± SEM (Cont mice, *n*=10; PI mice, *n*=7; PI+Ex mice, *n*=10). Data were analyzed using one-way ANOVA with Tukey’s *post hoc* test. **p* < 0.05, ***p* < 0.01 compared with the Cont mice and *^#^p* < 0.05 compared with PI mice.

#
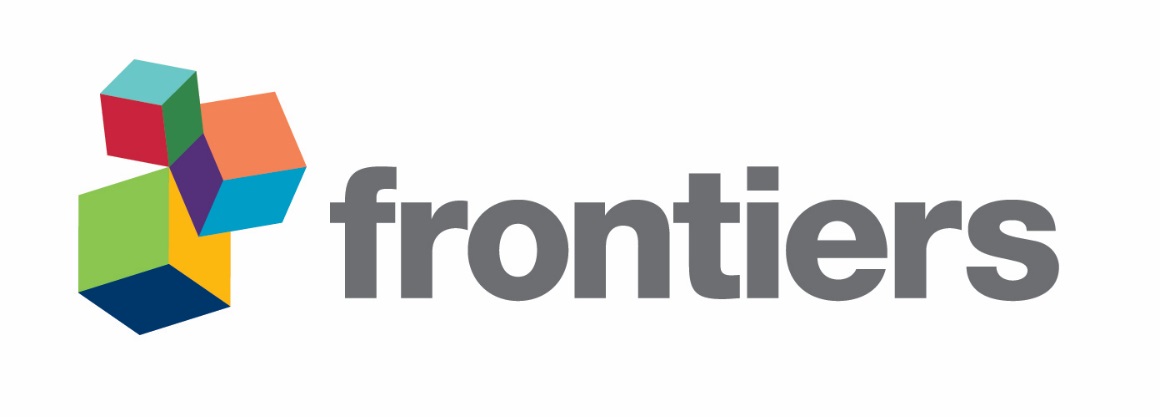

Supplement: Supplementary file 1 [file Table_1.docx]
